# Methylated 1,2-naphthoquinone derivative SJ006 as an inhibitor of human glucose 6-phosphate dehydrogenase in non-small cell lung cancer cell lines

**DOI:** 10.1038/s41598-025-20702-6

**Published:** 2025-10-21

**Authors:** Makamas Chanda, Warinthorn Chavasiri, Panupong Mahalapbutr, Thanyada Rungrotmongkol, Poonlarp Cheepsunthorn, Chalisa Louicharoen Cheepsunthorn

**Affiliations:** 1 Interdisciplinary Program of Biomedical Sciences, Graduate School, Chulalongkorn University, Bangkok, Thailand; 2 Department of Chemistry, Faculty of Science, Chulalongkorn University, Bangkok, Thailand; 3 Department of Biochemistry, Faculty of Medicine, Khon Kaen University, Khon Kaen, Thailand; 4 Department of Biochemistry, Faculty of Science, Chulalongkorn University, Bangkok, Thailand; 5 Department of Anatomy, Faculty of Medicine, Chulalongkorn University, Bangkok, Thailand; 6 Department of Biochemistry, Faculty of Medicine, Chulalongkorn University, 1873 Rama 4 Road, Pathumwan, Bangkok, 10330 Thailand

**Keywords:** Anticancer agent, Cytotoxicity, G6PD, Methylated 1,2-naphthoquinone, NSCLC, Uncompetitive inhibitor, Biochemistry, Cancer, Molecular biology, Medical research, Oncology

## Abstract

Glucose 6-phosphate dehydrogenase (G6PD) is crucial for redox balance and biosynthesis via the pentose phosphate pathway (PPP), driving non-small cell lung cancer (NSCLC) proliferation. This study assessed the cytotoxic and enzymatic effects of five 1,2-naphthoquinone (NQ) derivatives, including SJ006 derived from *Usnea barbata*, in NSCLC cell lines (A549 and NCI-H292) compared to traditional inhibitors (DHEA and 6AN). All 1,2-NQs demonstrated concentration-dependent cytotoxicity against NSCLC cells. Among them, NN02 exhibited the highest cytotoxicity comparable to 6-AN, followed by NN01, NN04, SJ006, and SJ007, which showed moderate effects comparable to DHEA. SJ006 uniquely inhibited G6PD activity without altering its mRNA or protein expression. Unlike DHEA and 6AN, SJ006 functioned as an uncompetitive inhibitor, decreasing both K_m_ and V_max_, with molecular docking confirming strong G6PD binding. Additionally, SJ006 increased reactive oxygen species (ROS) levels, induced G2/M cell cycle arrest, and triggered late apoptosis in NSCLC cells. Its effects were reversed by D-(−)-ribose, confirming PPP disruption as the mechanism. These results highlight SJ006 as a novel G6PD inhibitor that disrupts redox homeostasis and biosynthesis-driven cell proliferation, showing promise as an anticancer agent for NSCLC.

**Supplementary Information:**

The online version contains supplementary material available at 10.1038/s41598-025-20702-6.

## Introduction

The pentose phosphate pathway (PPP) is essential in cellular metabolism, generating ribose 5-phosphate (R5P) for nucleotide synthesis and producing nicotinamide adenine dinucleotide phosphate (NADPH), a key reducing agent for biosynthesis processes and antioxidant defense. Glucose 6-phosphate dehydrogenase (G6PD) (E.C.1.1.1.49)^1^, the rate-limiting enzyme in the oxidative branch of the PPP, plays a key role in cancer progression by promoting redox homeostasis and enhancing cell survival and proliferation in response to oxidative stress^2–6^. High G6PD activity has been linked to cancer aggressiveness, including metastasis, poor prognosis, and chemoresistance^7,8^, particularly in non-small cell lung cancer (NSCLC), the most prevalent form of lung cancer^9–13^.

Several classical G6PD inhibitors, including dehydroepiandrosterone (DHEA) and 6-aminonicotinamide (6-AN), have demonstrated potential anticancer effects through disruption of redox balance and metabolic reprogramming in tumor cells^14–17^. Although DHEA act as a noncompetitive G6PD inhibitor, it exhibits pleiotropic biological effects, including modulation of mitochondrial gene expression, steroidogenesis, and immune regulation that complicate attribution of its anticancer effects solely to G6PD inhibition^18–20^. Moreover, DHEA has poor pharmacokinetic properties, including rapid metabolism and extensive conversion to androgenic metabolites, which limits its specificity and raises concerns about off-target hormonal effects^6,21–27^. High-dose requirements and species-specific toxicities, including severe autoinflammatory responses in animal models, further restrict its clinical utility^6,21–27^.

Likewise, 6-AN inhibits G6PD competitively by mimicking NADP⁺, impairing NADPH production and increasing oxidative stress, which sensitizes cancer cells to chemotherapy and radiation^9,28^. However, its lack of target specificity and concurrent inhibition of other NADP^+^ - dependent enzymes, such as 6—phosphogluconate dehydrogenase, result in off-target cytotoxicity affecting normal cells^29,30^. Furthermore, its in vivo toxicity and limited pharmacokinetic data have prevented its advancement into clinical development^6,21–27^. To address these limitations, we investigated a new G6PD inhibitor with improved in vitro activity and target selectivity.

Quinone derivatives, including naphthoquinones (NQs), are promising anticancer agents due to their ability to induce oxidative stress and disrupt cellular redox balance^31,32^. The importance of quinone structures in cancer therapy is exemplified by doxorubicin, a widely used chemotherapeutic agent, contains a quinone moiety^33^. Among these, 1,2-naphthoquinone (1,2-NQ) derivatives have been extensively investigated for their diverse biology activities and anticancer properties^32,34^. This study focuses on five 1,2-NQ derivatives: mansonone G (NN01), its derivatives NN02 and NN04, SJ006, and β-lapachone (SJ007). These compounds have been explored for their structural modifications and potential as anticancer agents^35–37^. Mansonone G (NN01) serves as the parent compound for NN02 and NN04, featuring a naphthalene core with isopropyl and methyl groups^37^. NN02 and NN04 are prenyl and allyl ether derivatives of mansonone G, respectively, with modifications at the 6-position aimed at enhancing lipophilicity and biological activity^37^. As previously reported by Hairani et al. (2016), these derivatives exhibit improved cytotoxic effects, potentially by inducing oxidative stress and disrupting redox balance^37^. SJ006 and SJ007 are 1,2-NQ derivatives synthesized from lapachol, a natural quinonoid precursors^38^. SJ006 (2-methyl-2,3-dihydronaphtho[1,2-b]furan-4,5-dione) has demonstrated anticancer activity in human leukemia cell lines^35,36^. SJ007 (2,2-dimethyl-3,4-dihydro-2 H-benzo[h]chromene-5,6-dione), also known as β-lapachone, targets various cancer pathways and is currently in phase II clinical trials for advanced solid tumors^35,36^. The structural diversity and anticancer potential of these 1,2-NQ derivatives emphasize their significance as candidates for further research.

Understanding the precise mechanisms and molecular targets of anticancer compounds, particularly their ability to inhibit G6PD, can facilitate the development of innovative strategies to combat malignancies such as NSCLC. This study explores the anticancer potential of SJ006 by evaluating its cytotoxic concentration (CC_50_) and G6PD inhibitory effects in NSCLC cell lines. A549 and H292 cell lines were selected as representative models of NSCLC due to their distinct histological subtypes and differing G6PD activity profiles^9–13^, enabling a broader assessment of SJ006 efficacy across tumor heterogeneity. SJ006 was found to induce reactive oxygen species (ROS) production and exert antiproliferative effects, which were reversible upon the addition of D-(–)-ribose, highlighting its mechanism of action through inhibition of the G6PD-regulated pentose phosphate pathway (PPP). As an uncompetitive G6PD inhibitor, SJ006 induced G2/M-phase arrest and apoptosis without altering G6PD mRNA or protein expression levels, suggesting direct enzymatic inhibition rather than transcriptional regulation. Importantly, SJ006 was designed to overcome the key limitations of DHEA and 6-AN. It demonstrates enhanced cellular activity and selectivity toward G6PD, which may reduce off-target effects and toxicity. While these results are promising, further pharmacokinetic and in vivo studies are required to validate its potential for clinical application. Together, these findings position SJ006 as a promising candidate for G6PD-targeted cancers in NSCLC.

## Results

### **Characteristics and cytotoxic concentrations (CC**_**50**_**) of 1**,**2-NQ derivatives in NSCLC cell lines**

**Fig. 1:**
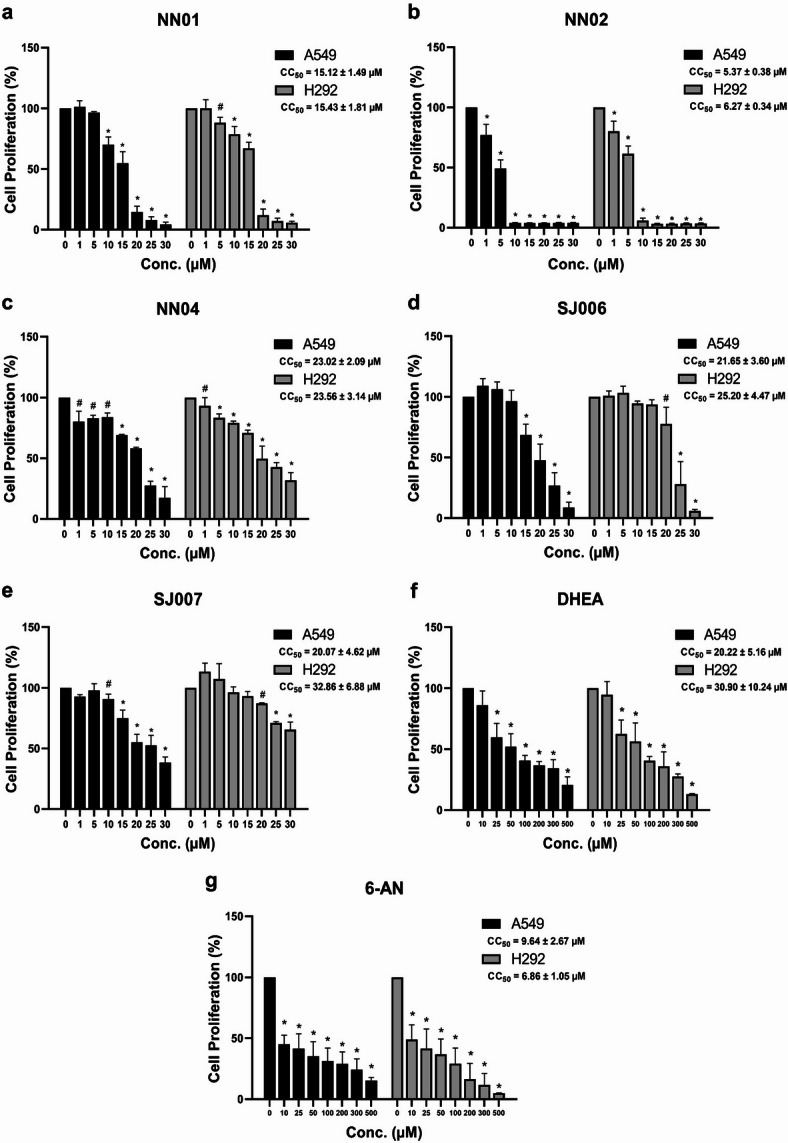
Cytotoxic concentration (CC_50_) of five 1,2-NQ derivatives (NN01, NN02, NN04, SJ006, and SJ007), DHEA, and 6-AN in A549 and H292 cells. (**a**) NN01, (**b**) NN02, (**c**) NN04, (**d**) SJ006, (**e**) SJ007, (**f**) DHEA, and (**g**) 6-AN in A549 and H292 cells. Data are presented as mean ± SEM (*n* = 3 independent experiments). Statistical significance versus control (0 µM) was determined by one‑way ANOVA followed by LSD post‑hoc test; ^#^*p* < 0.05 and *p < 0.001.

The characteristics of five 1,2-NQ derivatives, including NN01, NN02, NN04, SJ006, and SJ007, are summarized in Table 1. The cytotoxic effects (CC_50_) of these derivatives, along with the known inhibitors DHEA and 6-AN, were assessed in A549 and H292 cells following 48 h of treatment, revealing concentration-dependent effects (Fig. 1a-g). Among the tested compounds, NN02 exhibited the highest cytotoxicity, with CC_50_ values of 5.37 ± 0.38 µM in A549 cells and 6.27 ± 0.34 µM in H292 cells, comparable to 6-AN (CC_50_: 9.64 ± 2.67 µM in A549 cells, CC_50_: 6.86 ± 1.05 µM in H292 cells). NN01 displayed intermediate cytotoxicity, with CC_50_ values of 15.12 ± 1.49 µM in A549 cells and 15.43 ± 1.81 µM in H292 cells. In contrast NN04 (CC_50_: 23.02 ± 2.09 µM in A549 cells, CC_50_: 23.56 ± 3.14 µM in H292 cells), SJ006 (CC_50_: 21.65 ± 3.60 µM in A549 cells, CC_50_: 25.20 ± 4.47 µM in H292 cells), and SJ007 (CC_50_: 20.07 ± 4.62 µM in A549 cells, CC_50_: 32.86 ± 6.88 µM in H292 cells) demonstrated comparatively lower cytotoxic effects, comparable to DHEA (CC_50_: 20.22 ± 5.16 µM in A549 cells and CC_50_: 30.90 ± 10.24 µM in H292 cells). Notably, the CC_50_ values for SJ007 in H292 cells could not be determined, as cell viability plateaued at approximately 60% at a concentration of 30 µM, indicating reduced sensitivity to this compound compared to the other derivatives. These results highlight the differential cytotoxic potential of the 1,2-NQ derivatives and the reference inhibitors against NSCLC cell lines.

**Table 1:**
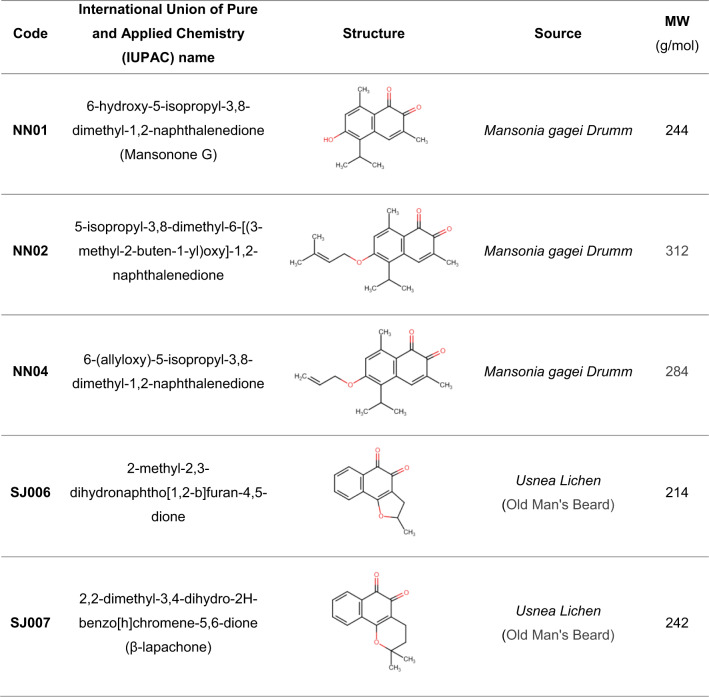
Characteristics, including the international union of pure and applied chemistry name (IUPAC), structure, sources, and molecular weight (MW) of five 1,2-NQ derivatives (NN01, NN02, NN04, SJ006, and SJ007).

## Inhibitory effect of 1, 2-NQ derivatives on G6PD activity

**Fig. 2:**
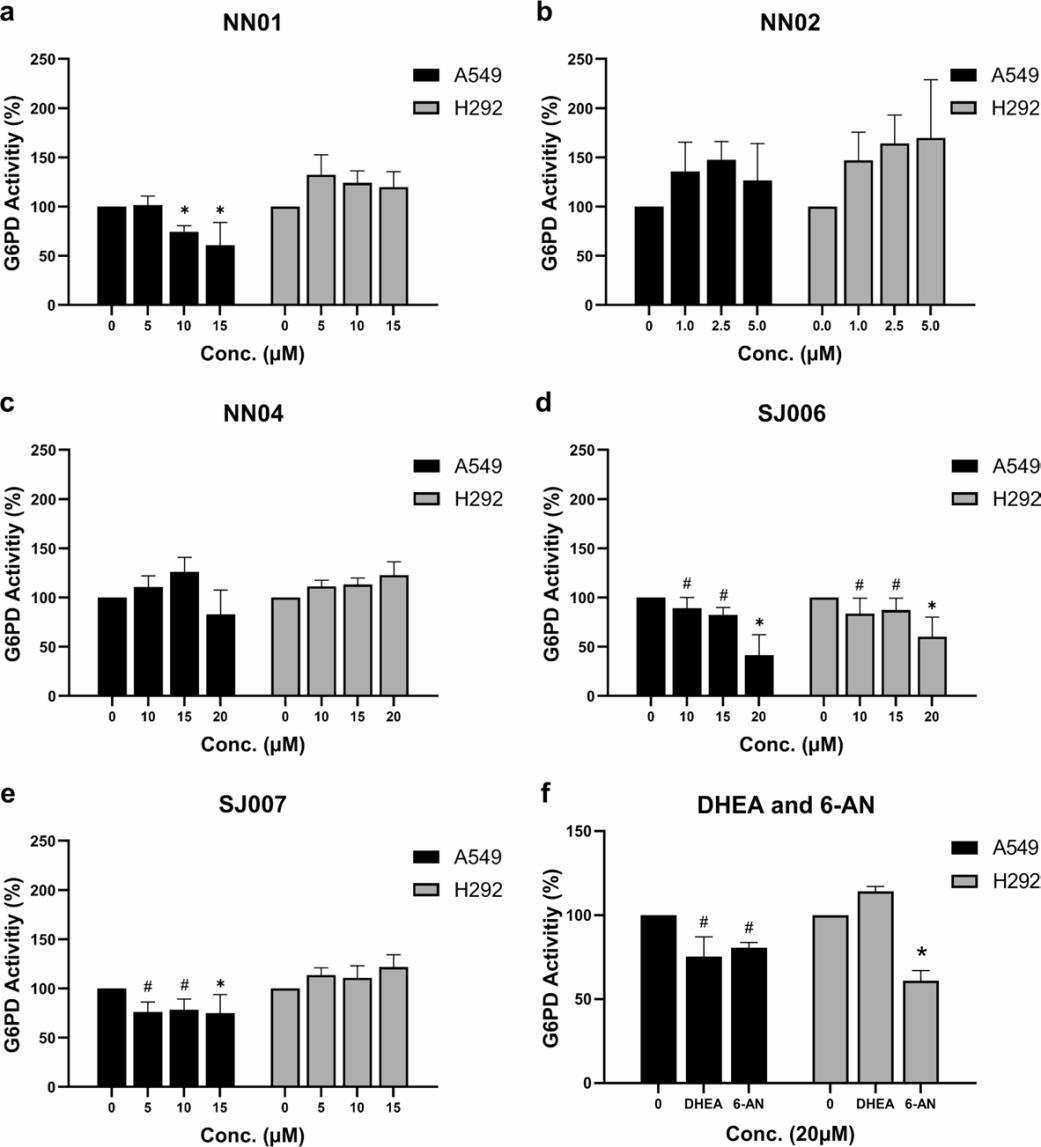
Inhibitory effects of 1,2-NQ derivatives and known G6PD inhibitors on G6PD activity in A549 and H292 cells. (**a**–**e**) G6PD activity inhibition by NN01, NN02, NN04, SJ006, and SJ007. (**f**) Comparative inhibition of G6PD activity by DHEA and 6-AN. Data are presented as mean ± SEM (*n* = 3 independent experiments). Statistical significance versus control (0 µM) was determined by one‑way ANOVA followed by LSD post‑hoc test; ^#^*p* < 0.05 and ^*^p < 0.001.

The inhibitory effects of five 1,2-naphthoquinone (1,2-NQ) derivatives and known G6PD inhibitors (DHEA and 6-AN) on G6PD activity were evaluated following treatment at non-toxic concentrations (below CC_50_ values) for 48 h. As shown in Fig. 2a–f, SJ006 significantly inhibited G6PD activity in a concentration-dependent manner in both A549 and H292 cell lines. NN01 and SJ007 also reduced G6PD activity, but only in A549 cells, whereas NN02 and NN04 exhibited no inhibitory effects on G6PD activity in both cell lines. Among the reference inhibitors, 6-AN (20 µM) effectively inhibited G6PD activity in both NSCLC cell lines, whereas DHEA (20 µM) reduced G6PD activity only in A549 cells.

**Fig. 3:**
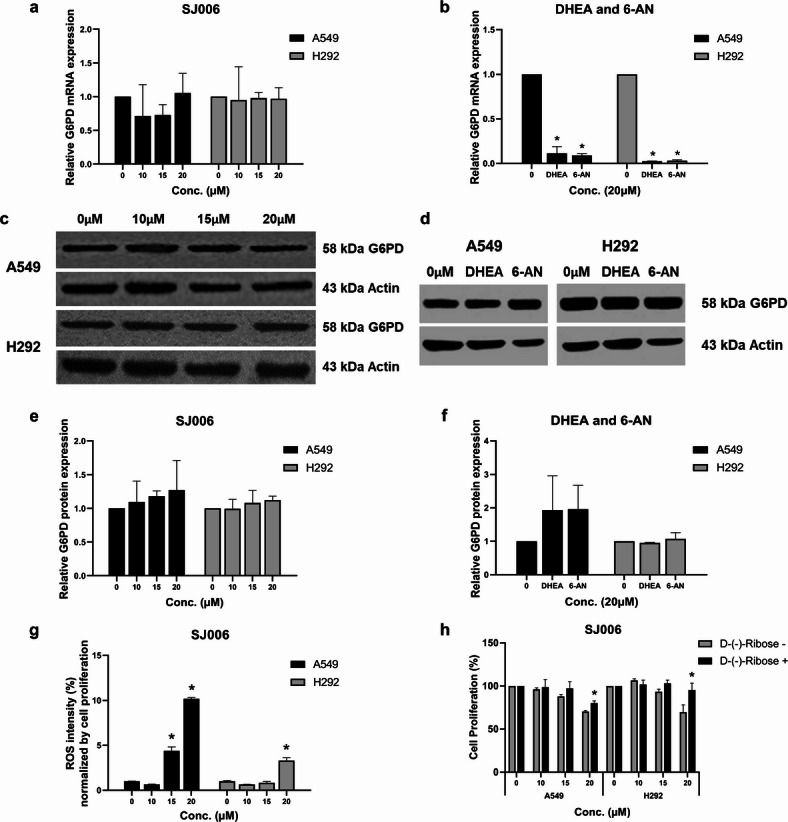
Effects of SJ006 on G6PD expression, redox homeostasis, and cell viability in A549 and H292 cells. (**a**) Relative G6PD mRNA expression after treatment with SJ006, and (**b**) DHEA and 6-AN. G6PD protein expression (Western blot: WB) following treatment with (**c**) SJ006, and (**d**) DHEA, and 6-AN. Relative G6PD protein levels following treatment with (**e**) SJ006, and (**f**) DHEA and 6-AN. (**g**) Induction of ROS after SJ006 treatment. (**h**) Cell viability after treatment with SJ006 followed by rescue with D-(−)-ribose. Data are presented as mean ± SEM (*n* = 3 independent experiments). Statistical analysis was performed using one‑way ANOVA followed by LSD post‑hoc test for SJ006‑treated groups, and one‑sample t‑test for DHEA and 6‑AN treatments. Significant differences compared to the control (0 µM) are denoted as ^*^p < 0.001.

To investigate whether SJ006’s inhibition of G6PD activity was due to direct enzymatic inhibition or changes in G6PD expression, we measured G6PD mRNA and protein levels. SJ006 treatment did not affect G6PD mRNA expression in both cell lines (Fig. 3a). In contrast, both DHEA and 6-AN treatment significantly reduced G6PD mRNA expression (Fig. 3b). However, G6PD protein levels were not altered by treatment with SJ006, DHEA, or 6-AN in both cell lines (Fig. 3c–f, Supplementary Fig. S1). Notably, in H292 cells treated with DHEA, G6PD mRNA levels were significantly reduced; however, this was not accompanied by a decrease in G6PD protein levels or enzyme activity. This discrepancy is likely due to the relative stability and long half-life of the G6PD protein, which can sustain enzymatic activity despite transcriptional downregulation. The unchanged activity also suggests that DHEA does not directly impair G6PD enzymatic function or post-translational regulation but primarily affects gene transcription. Since mRNA, protein, and activity were measured at the same time point, it is possible that a delayed reduction in protein abundance and activity may occur at later time points, reflecting the observed decrease in mRNA expression.

To further investigate the impact of G6PD inhibition by SJ006 on redox homeostasis, we assessed ROS levels following treatment. SJ006 treatment significantly increased ROS levels in NSCLC cells (Fig. 3g). In A549 cells, treatment with 20 µM SJ006 elevated ROS levels by 10.19-fold compared to the control. Similarly, in H292 cells, SJ006 treatment resulted in a 3.3-fold increase in ROS levels relative to the control.

Finally, to confirm the involvement of the oxidative branch of the PPP in SJ006’s mechanism of action, cells were pretreated with D-(−)-Ribose, a downstream product of the PPP. Pretreatment with D-(−)-Ribose partially rescued cell viability in A549 and H292 cells treated with SJ006, although this rescue effect was not significant at lower concentrations of SJ006 (Fig. 3h).

## SJ006 as an uncompetitive G6PD inhibitor

**Fig. 4:**
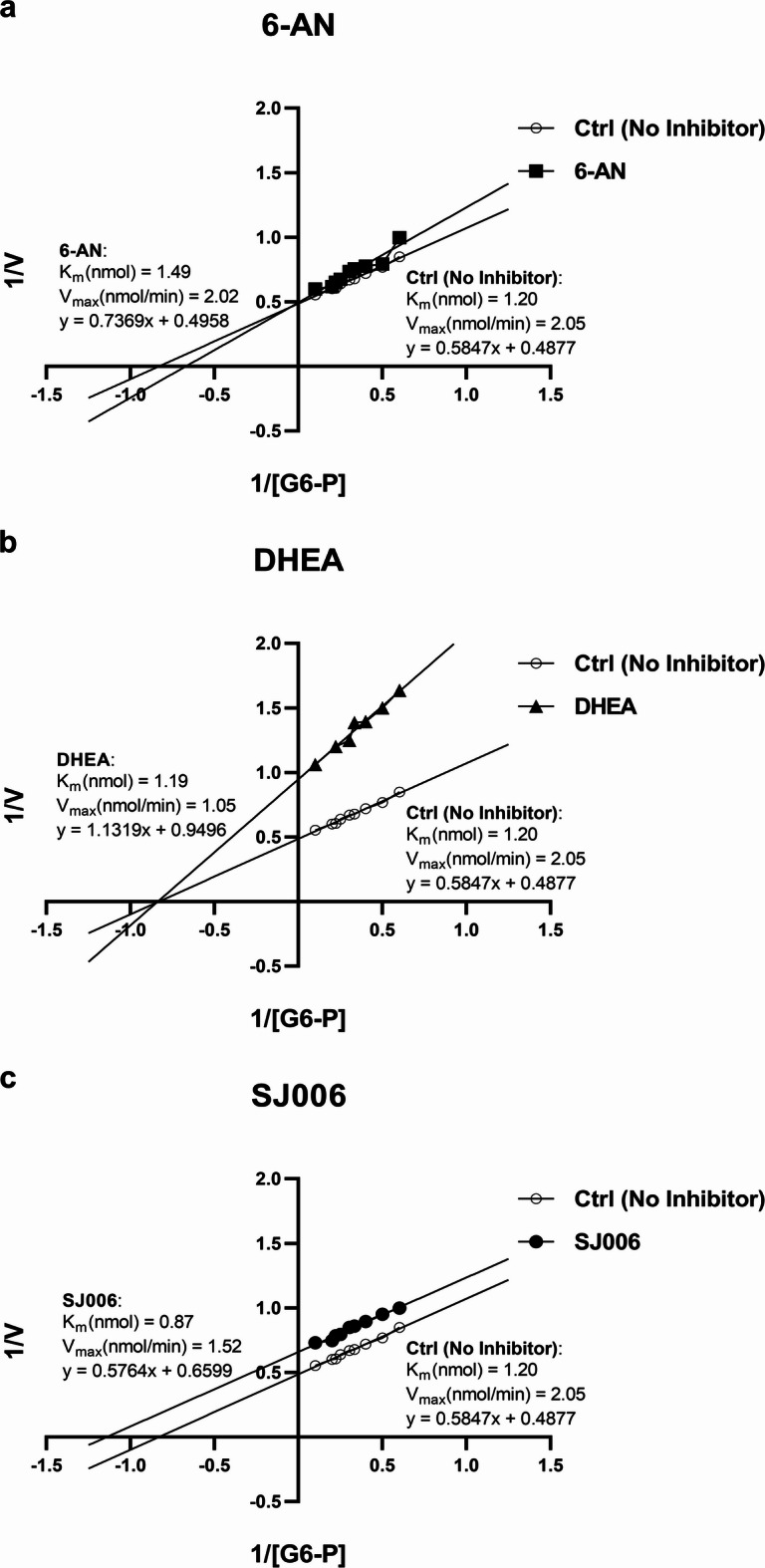
Lineweaver–Burk plots and corresponding K_m_ and V_max_ values for G6PD activity inhibition by each G6PD inhibitor: (**a**) 6-AN, (**b**) DHEA, and (**c**) SJ006. The plots illustrate the distinct inhibitory mechanisms of the compounds, with 6-AN acting as a competitive inhibitor, DHEA as a noncompetitive inhibitor, and SJ006 as an uncompetitive inhibitor.

This study aimed to characterize the inhibitory mechanism of SJ006 on G6PD and compare it with established inhibitors 6-AN and DHEA using Michaelis–Menten analysis and Lineweaver–Burk plots, providing insights into its distinct molecular interaction. Consistent with prior reports, 6-AN acted as a competitive inhibitor, characterized by an increase in K_m_ while V_max_ remained unchanged^29^ (Fig. 4a). DHEA, in contrast, exhibited noncompetitive inhibition, which reduced V_max_ without altering K_m_^29,39^ (Fig. 4b). SJ006 was identified as an uncompetitive inhibitor, as evidenced by a simultaneous reduction in both K_m_ and V_max_ (Fig. 4c).

## Molecular docking analysis and two-dimensional (2D) interaction profiles of SJ006 with G6PD

**Fig. 5:**
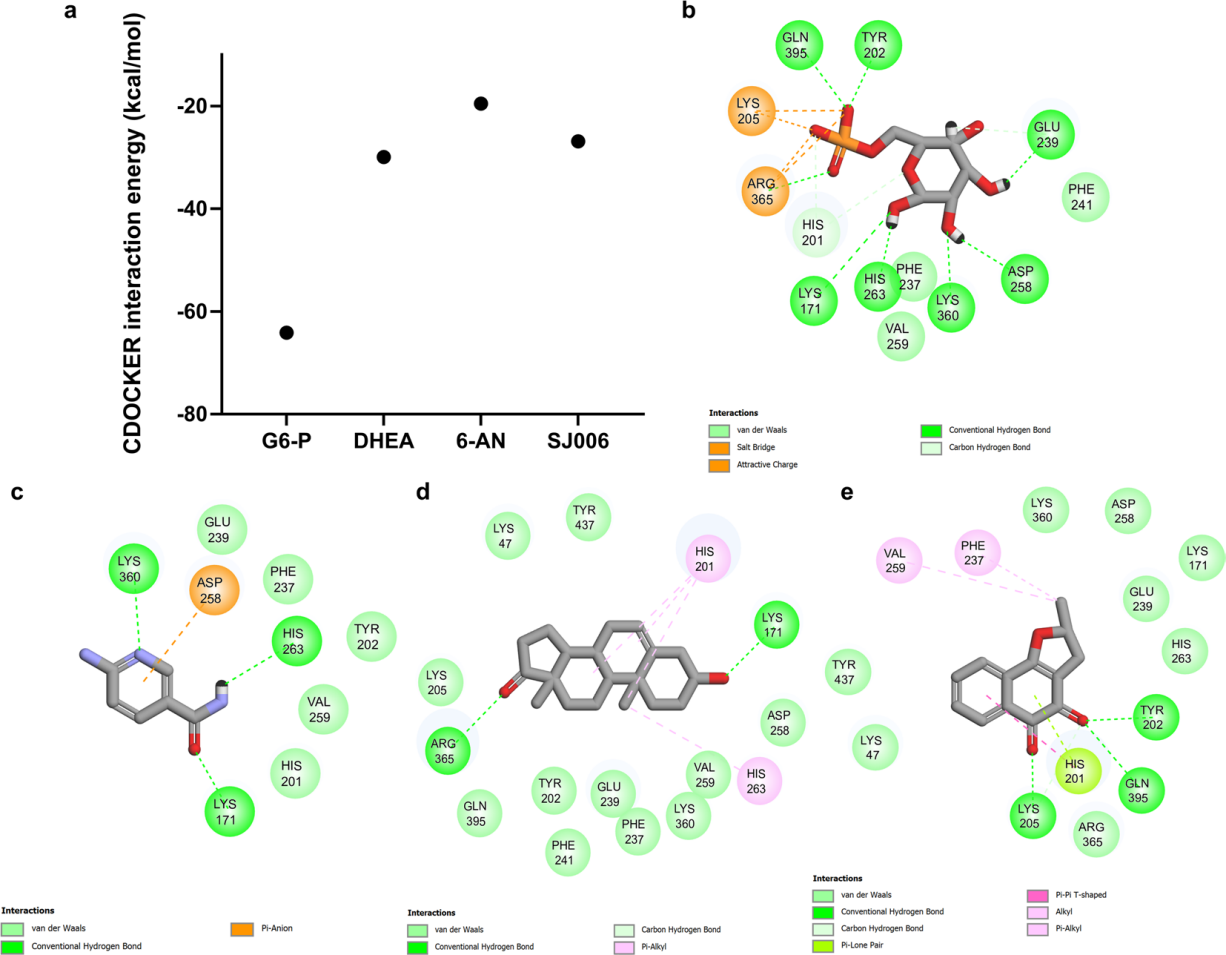
Molecular docking energies and 2D interaction profiles of G6PD with SJ006, G6-P (the natural substrate for G6PD), DHEA, and 6-AN. (**a**) Comparison of molecular docking energies of SJ006, G6-P, DHEA, and 6-AN. 2D interaction profiles illustrating binding interactions of G6PD with (**b**) G6-P, (**c**) 6-AN, (**d**) DHEA, and (**e**) SJ006, highlighting key residues involved in hydrogen bonding, van der Waals forces, alkyl interactions, and other binding interactions.

Molecular docking analysis revealed that SJ006 exhibited stronger binding affinity to G6PD compared to 6-AN and an affinity comparable to DHEA (Fig. 5a). The 2D interaction profiles of the ligand-protein complexes indicated that SJ006 interacted with 14 G6PD residues, surpassing the 9 residues engaged by 6-AN and approaching the 15 residues involved in the DHEA complex (Fig. 5b–e). The SJ006/G6PD complex was stabilized by robust van der Waals and alkyl interactions, which were more pronounced than those observed in the 6-AN/G6PD complex. Although the binding affinity of SJ006 was comparable to DHEA, SJ006 exhibited a more diverse interaction profile, including Pi-stacking interactions, which may contribute to a stronger and more stable binding than DHEA. Notably, SJ006 established specific interactions with G6PD residues, including hydrogen bonds (Tyr202, Lys205, Asp258, Arg365, and Gln395), salt bridges and pi-cation/anion interactions (Glu239 and Lys360), and pi-lone pair interactions (His201) (Fig. 5e). These results highlight the potential of SJ006 as a G6PD inhibitor, though its binding affinity remains lower than that of the enzyme’s natural substrate (Fig. 5b). Despite these strong interactions observed for SJ006, the binding affinity of G6-P for G6PD surpassed that of all tested compounds. This superior affinity was attributed to its ability to form a greater number of hydrogen bonds and salt bridges formed in the G6-P/G6PD complex. Molecular docking analysis and the 2D interaction profiles of other 1,2-NQs derivatives-G6PD complexes were shown in Supplementary Fig. S2.

## Effect of SJ006 on cell cycle arrest and apoptosis

**Fig. 6:**
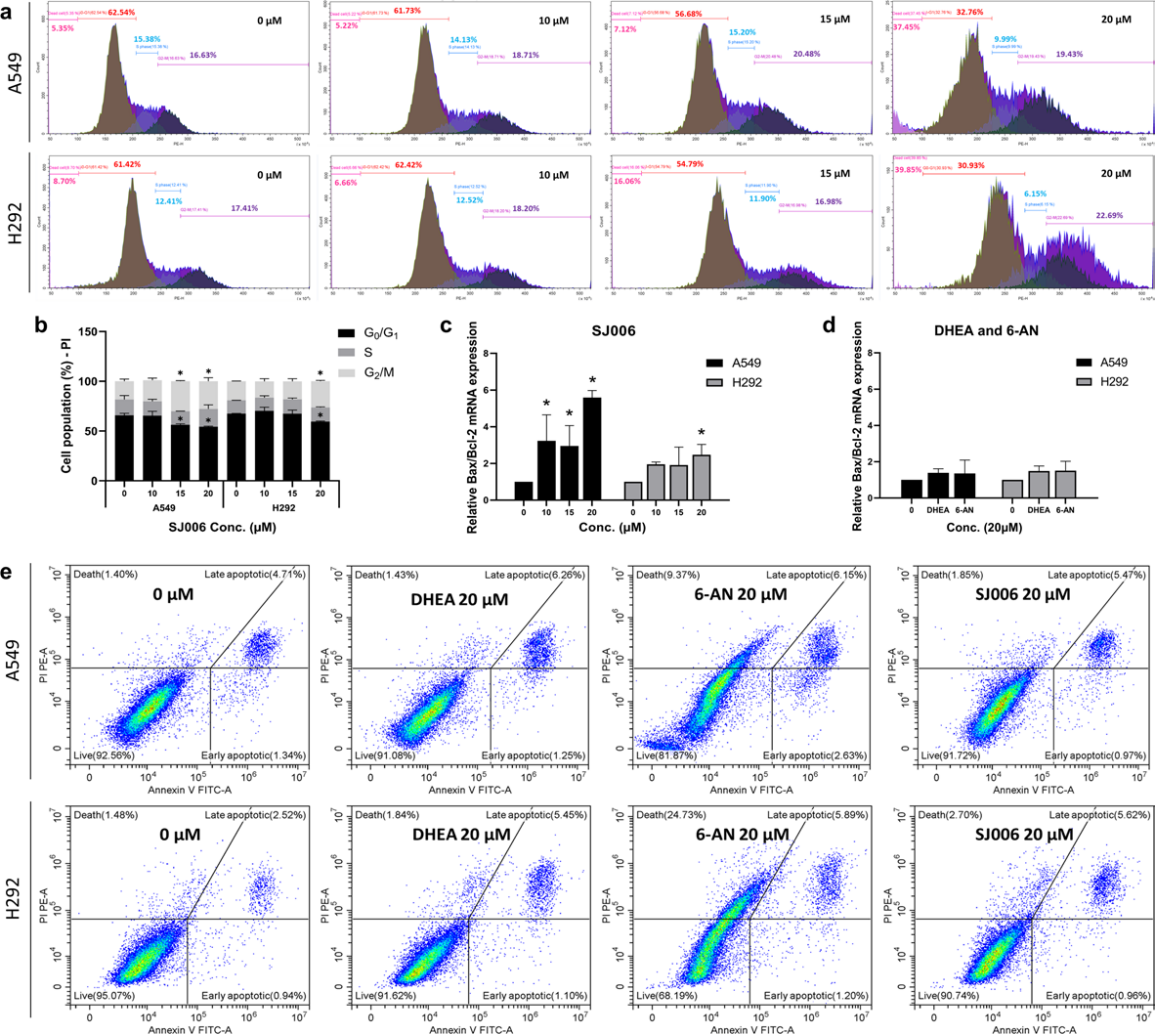
Effects of SJ006 on cell cycle arrest and apoptosis by G6PD inhibition in A549 and H292 cells. SJ006 induced (**a**, **b**) G2/M-phase cell cycle arrest, and (**c**) activated apoptosis markers, evidenced by changes in Bax/Bcl2 mRNA expression, compared to (**d**) DHEA and 6-AN. (**e**) Apoptosis analysis using annexin V/PI staining revealed distinct cell populations: viable cells (lower left quadrant), early apoptotic cells (lower right quadrant), necrotic cells (upper left quadrant), and late apoptotic cells (upper right quadrant) for control (0 µM), DHEA, 6-AN, and SJ006 in A549 and H292 cells. Data are presented as mean ± SEM (*n* = 3 independent experiments). Statistical analysis was performed using one‑way ANOVA followed by LSD post‑hoc test for SJ006-treated groups, and one‑sample t‑test for DHEA and 6‑AN treatments. Significant differences compared to control (0 µM) are denoted as ^#^*p* < 0.05 and *p < 0.001.

The impact of G6PD inhibition by SJ006 on cell cycle progression and apoptosis was assessed in A549 and H292 cells. SJ006 treatment at 20 µM induced cell cycle arrest, reducing the G0/G1 phase population (by 11.52% and 7.97% in A549 and H292 cells, respectively) while increasing the G2/M-phase population (by 9.50% and 7.22%, respectively) compared to control cells (Fig. 6a, b).

Apoptotic effects were analyzed by evaluating the Bax/Bcl-2 mRNA ratio, a marker of apoptosis induction. SJ006 at 20 µM significantly elevated the Bax/Bcl-2 ratio in A549 and H292 cells (5.59-fold and 2.47-fold relative to control, respectively) (Fig. 6c**)**. In contrast, DHEA and 6-AN treatment did not significantly alter Bax/Bcl-2 mRNA expression (Fig. 6d).

Further analysis using Annexin V/PI assays confirmed the pro-apoptotic effects of SJ006. At 20 µM, SJ006 markedly induced late apoptosis in A549 and H292 cells (1.14-fold and 1.92-fold relative to control, respectively), accompanied by a reduction in viable cells in A549 and H292 cells (0.95-fold and 0.99-fold relative to control, respectively). Similarly, DHEA increased late apoptosis in both A549 and H292 cells (1.18-fold and 1.80-fold relative to control, respectively). Treatment with 6-AN induced both early apoptosis (1.23-fold and 1.28-fold relative to control, respectively) and late apoptosis (1.04-fold and 1.93-fold relative to control, respectively), along with an increase in necrosis in both A549 and H292 cells (7.69-fold and 18.80-fold relative to control, respectively) (Fig. 6e) (Supplementary Fig. S3).

## Discussion

Lung cancer is the leading cause of cancer-related deaths globally, with a 5-year survival rate of less than 18%^11^. G6PD, a pivotal enzyme in the PPP, is overexpressed in lung cancers, particularly in NSCLC tissues and cisplatin-resistant cell lines, contributing to chemoresistance, poor survival outcomes, and relapse^9,10,12,13^. By providing macromolecules and NADPH for proliferation and redox balance, G6PD is essential for cancer cell metabolic reprogramming^40^. Targeting the PPP through G6PD inhibition offers a promising therapeutic strategy and a focus for novel inhibitor development^2,21,40–43^. This study explored the cytotoxic effects of 1,2-NQ derivatives in NSCLC cell lines and demonstrated their potential to disrupt G6PD activity, oxidative stress, and metabolism, positioning them as targeted anticancer agents.

Among the derivatives, mansonone G (NN01) is a natural quinone derived from the heartwood of *Mansonia gagei*, exhibiting anticancer effects primarily through the generation of reactive oxygen species (ROS), leading to apoptosis and cell cycle arrest^39^ NN01 has shown significant potential in NSCLC models by inducing cell death through apoptosis pathways regulated by caspase enzymes and affecting signaling pathways like STAT3, Akt, and Erk^39^. Consistent with previous findings, our results confirm the cytotoxic effects of mansonone G on NSCLC proliferation. Interestingly, its inhibitory effect on G6PD activity was observed in adenocarcinoma human alveolar basal epithelial cells (A549) but not in human lung mucoepidermoid carcinoma cells (NCI-H292). This discrepancy may stem from the concentration used in H292 cells being insufficient to inhibit G6PD activity, as H292 cells exhibit a higher basal level of G6PD compared to A549 cells^13^.

NN02 (5-Isopropyl-3,8-dimethyl-6-[(3-methyl-2-buten-1-yl)oxy]-1,2-NQ) and NN04 (6-(allyloxy)-5-isopropyl-3,8-dimethyl-1,2-NQ) are synthetic quinone derivatives with limited prior characterization regarding anticancer activity. In this study, NN02 demonstrated the highest cytotoxicity, while NN04 effectively inhibited NSCLC proliferation. Unlike mansonone G, their mechanisms appear independent of G6PD inhibition in both cells, suggesting structure-specific actions. Differences in structural characteristics and physicochemical properties among NN01, NN02, and NN04 likely contribute to their differential effects on G6PD activity, which appear to be concentration-dependent^42^.

SJ007 (β-Lapachone or 2,2-dimethyl-3,4-dihydro-2 H-benzo[h]chromene-5,6-dione) is the most extensively studied among these 1,2-NQ derivatives. It exhibits potent anticancer effects, particularly in NAD(P)H quinone dehydrogenase 1 (NQO1)-overexpressing cancer cells, a characteristic commonly found in NSCLC^44^. SJ007 undergoes NQO1-mediated redox cycling, leading to excessive ROS production, DNA damage, and hyperactivation of poly(ADP-ribose) polymerase (PARP), culminating in cancer cell death^44^. The use of SJ007 (β-lapachone analogue or ARQ 761) in a clinical setting is under investigation, including its combination with standard chemotherapeutic agents like gemcitabine and nab-paclitaxel for advanced pancreatic cancer^44,45^. Despite its well-documented ROS-generating mechanism, our findings revealed that SJ007 exhibited relatively low cytotoxicity in NSCLC, particularly in H292 cells. We also observed that SJ007 inhibited G6PD activity in A549 cells but not in H292 cells, suggesting possible differences in redox metabolism or NQO1 expression between these cell lines that may modulate its efficacy. Overall, while not all derivatives acted through G6PD inhibition, our results reveal the diverse and compound-specific nature of action among this class, underscoring the broader therapeutic potential of these derivatives beyond G6PD targeting. This highlights the need for further studies to comprehensively elucidate the molecular targets and mechanisms of action of these promising compounds. However, we firstly focused on G6PD inhibition due to its pivotal role in redox homeostasis and cancer metabolism, particularly in NSCLC.

In contrast, SJ006 (2-Methyl-2,3-dihydronaphtho[1,2-b]furan-4,5-dione), a naphthoquinone-furan derivative from *Usnea lichens*, has been explored for its potential anticancer properties. Previous studies have reported the antiproliferative effect of SJ006 on leukemia cells (HL-60)^35,36^. Our findings support these results, demonstrating that SJ006 effectively reduces NSCLC cell proliferations by directly inhibiting G6PD activity. Unlike other inhibitors such as DHEA and 6-AN, SJ006 does not affect G6PD mRNA or protein expression, indicating that its mechanism is independent of transcriptional regulation. In comparison, DHEA failed to significantly inhibit G6PD activity in H292 cells at 20 µM in our study, a concentration chosen for its physiological relevance and to match SJ006 exposure. The inhibitory effect of DHEA on G6PD activity is known to be dose-dependent and varies across cell types. Previous studies suggest that DHEA requires higher doses (around 300 µM) to inhibit G6PD in H292 cells^13^, underscoring its limited therapeutic window. Furthermore, DHEA’s pleiotropic actions and cell-specific redox adaptations may reduce its effectiveness in certain cancer contexts. Given the observed non-specific inhibitory effects of 6-AN and DHEA on various cellular processes and targets^19,20,29,30^, further investigation is needed to determine whether SJ006 exerts off-target effects that contribute to its anticancer activity beyond G6PD inhibition.

The inhibition of G6PD by SJ006 disrupts redox homeostasis and depletes nucleotide precursors critical for cell proliferation. Our results indicate that ROS production increases upon SJ006 treatment, highlighting the compound’s role in disturbing redox balance. NSCLC cell proliferation was partially rescued by D-(−)-Ribose, an end product of the PPP and a precursor for nucleotide synthesis. However, ribose does not directly rescue the inhibitory effect of SJ006 on G6PD activity. Instead, ribose supports cell proliferation by providing nucleotides necessary for DNA replication during the S phase of the cell cycle. While SJ006 inhibits G6PD activity, impacting cellular metabolism and redox balance, ribose aids in nucleotide biosynthesis, enabling the cells to progress through the cell cycle despite G6PD inhibition. This mechanism illustrates how ribose mitigates the effects of SJ006 on cell proliferation without directly competing for enzyme binding. These findings highlight the potential of SJ006 to selectively target the oxidative branch of the PPP. Further analysis of SJ006 revealed its impact on the cell cycle and apoptosis. SJ006 reduced the G0/G1 phase, increased G2/M arrest, and upregulated the Bax/Bcl-2 mRNA ratio, promoting late apoptosis and necrosis in A549 and H292 cells. In comparison, 6-AN induced both early and late apoptosis and significantly triggered necrosis. However, to our knowledge, 6-AN has not been previously reported to induce necrosis in cancer cells. Previous research on phenolic compounds from *Usnea* lichens demonstrated increased ROS production, DNA damage, G2/M arrest, and apoptosis^46–53^. These effects, coupled with the activation of JNK and p38 signaling pathways^54,55^, support SJ006’s role in ROS-mediated apoptosis and cell cycle arrest, consistent with mechanisms proposed for other 1,2-NQ derivatives^32,34^.

This study presents SJ006 as a novel uncompetitive G6PD inhibitor, reducing both the K_m_ and V_max_ of G6PD. Molecular docking analyses provide valuable insights into the protein-ligand interaction, facilitating in the identification of novel anticancer agents^37^. This suggests that SJ006 promotes tight G6PD-substrate binding, effectively preventing enzyme activity and potentially altering the G6PD protein’s secondary structure^42^. These analyses indicated that SJ006 exhibits stronger binding affinity to G6PD compared to DHEA and 6-AN^14^. SJ006 forms a diverse range of interactions, including Pi-stacking, Pi-Alkyl interactions, and hydrogen bonds, whereas DHEA and 6-AN mainly rely on hydrogen bonds, which may limit their binding stability and specificity. These differences suggest that SJ006 might exhibit higher potency in inhibiting G6PD than DHEA and 6-AN. The binding energy of competitive, noncompetitive, and uncompetitive inhibitors differ because of their different mechanisms of action. Competitive inhibitors bind to the active site of the free enzyme, blocking substrate access. Noncompetitive inhibitors bind to an allosteric site, which is a location on the enzyme other than the active site, altering the enzyme’s function without directly competing with the substrate. In contrast, uncompetitive inhibitors, like SJ006, bind to the enzyme-substrate complex, further stabilizing it and preventing enzyme activity.

Overall, the observed antiproliferative and pro-apoptotic effects of SJ006 highlight its potential as an anticancer agent for NSCLC. However, limitations in this study include the lack of in vivo validation and exploration of potential adverse effects. Future research should focus on evaluating the efficacy and safety of SJ006 in animal models and its broader applicability across various cancer types to fully establish its potential as an anticancer agent.

## Methods

### Cells lines

Human non-small cell lung cancer (NSCLC) cell lines, NCI-H292 (mucoepidermoid carcinoma; ATCC No. CRL-1848) and A549 (adenocarcinoma; ATCC No. CCL-185) were selected based on our previous findings demonstrating that G6PD activity plays a pivotal role in NSCLC carcinogenesis^9–13^. A549 and H292 represent distinct histological subtypes of NSCLC and differ in their redox metabolism and baseline G6PD expression. Cells were cultured in Dulbecco’s Modified Eagle’s Medium supplemented with 10% (v/v) fetal bovine serum (Gibco), and 1% penicillin–streptomycin (v/v). Cells were maintained in a humidified incubator at 37 °C with 5% CO_2_ in 95% air, and the culture medium was replaced every 3 days.

### Compounds and molecular docking

NN01 (Mansonone G) was extracted and isolated from the dried heartwood of *Mansonia gagei* Drumm following previously published procedures^56,57^. NN02 and NN04 were synthesized from NN01 as previously described^37^. SJ007 were synthesized from lapachol, which was extracted from the lichen *Usnea barbata* (old man’s beard), and SJ006 was chemically modified from SJ007. All compounds were prepared by the team of Dr. Chavasiri W.

All compounds were determined to be > 95% purity by ^1^H and ^13^C NMR and analytical TLC prior to biological testing^37,57–60^. All compounds were stored in the dark at room temperature under low humidity until use. Stock solutions (10 mM) were prepared by dissolving the compounds in cell culture-grade DMSO and stored at 4 °C prior to experiments.

The crystal structure of human G6PD was obtained from the Protein Data Bank (PDB ID 2BHL). The three-dimensional structure of 1,2-NQ derivatives and G6PD inhibitors, including DHEA and 6-AN, was constructed using GaussView. The protein-ligand complexes were generated using CDOCKER module in Accelrys Discovery Studio 2.5 with 100 docking runs. The G6-P was defined as binding site with a docking sphere radius of 6.5 Å.

### Cytotoxic concentration (CC_50_) and cell viability assay

Cells were seeded into 96-well plates at a density of 5 × 10^3^ cells per well and incubated overnight. To determine the CC_50_ of 1,2-NQ derivatives, various concentrations were applied. After 48 h of treatment, cells were incubated with 25 µL of 2 mg/ml 3-(4,5-dimethylthiazol-2-yl)-2,5-diphenyltetrazolium (MTT) solution at 37 °C for 2 h. The MTT- solution was then removed, and the formazan dye formed by viable cells was dissolved in 75 µL of DMSO. Absorbance was measured at 570 nm using a microplate reader. All experiments were performed in triplicate, and cell viability was calculated as a percentage of the control (without NQ derivative).

To assess the inhibitory effect of 1,2-NQ derivatives, D-(−)-ribose (Sigma-Aldrich, catalog No. R9629) was dissolved in culture medium and filtered through a 0.22 μm membrane. After adding the sterile 5 mM D-(−)-ribose-containing medium, cells were incubated for 24 h, followed by treatment with the 1,2-NQ derivative for 48 h.

### G6PD activity assay, inhibitor test, and Western blotting

Cells were seeded into 25 cm^2^ canted-neck flasks at density of 8.75 × 10^4^ cells per flask and incubated for 24 h. The cells were then treated with 1,2-NQ derivatives at concentrations below the CC_50_. NQ derivatives also exhibited remarkable antiproliferative activity in colorectal adenocarcinoma cells (HT-29), with effective concentrations when CC_50_ was less than 20 µM^61^. After 48 h of treatment, cells were trypsinized (Trypsin, HyClone), and trypsinized cell pellets were collected by centrifugation. The pellets were washed with 1× PBS and sonicated to prepare lysates. Protein concentrations in the lysates were determined using the Pierce bicinchoninic acid (BCA) Protein Assay Kit.

We mixed 10 µL of cell lysates with 490 µL of G6PD reaction buffer, as described previously^62^. NADPH production was measured kinetically at 340 nm each min for 15 min at 37 °C using a Synergy HT microplate reader (BioTek instruments). G6PD activity was calculated by referencing a NADPH standard curve, with the absorbance values converted to units of NADPH production per min per mg protein to determine the CC_50_ of each compound. To determine K_m_ and V_max_ values and identify the type of inhibition, kinetic studies were performed with various concentrations of G6-P as previously described^62^. Lineweaver–Burk plots were generated to analyze the K_m_ and V_max_ in the presence of 6-AN, DHEA, and SJ006, which helped to determine the inhibition type.

We loaded 30 µg of protein from the cell lysates into individual wells of a stacking gel above a 10% SDS-containing polyacrylamide gel for electrophoresis (SDS–PAGE). The proteins were then transferred onto a nitrocellulose membrane by western blotting. After blocking nonspecific binding sites with 5% nonfat milk in Tris-buffered saline with 1% Tween 20 (TBST) for 1 h, the membranes were then incubated overnight at 4 °C with rabbit anti-G6PD antibodies (Sigma-Aldrich catalog No. HPA000834, Antibody Registry ID. AB_1078977) in TBST at a 1:2,500 dilution. Following three times for 5 min wash in TBST, the membranes were incubated for 2 h at room temperature with horseradish peroxidase-conjugated anti-rabbit secondary antibody. After three additional 5-min washes in TBST, G6PD immunoreaction was detected using an enhanced chemiluminescence (ECL) system (ThermoFisher Scientific) and visualized with a UVP ChemStudio PLUS Western Blot imaging system (Analytik Jena). The intensity of immunoreactive bands was quantified using VisionWorks software (Analytik Jena). Signal normalization was performed using β-actin (Sigma-Aldrich catalog No. A5441, Antibody Registry ID. AB_476744) as an internal control, with mouse anti-β-actin antibodies (Sigma-Aldrich catalog No. A9917, Antibody Registry ID. AB_258476).

### Quantitative real-time PCR (qRT-PCR)

Total RNA was isolated using Trizol reagent and quantified with a Nanodrop 1000 spectrophotometer (Thermo Fisher Scientific). Real-time PCR was performed to assess G6PD, Bax, and Bcl-2 mRNA expression. RNA was reverse transcribed into cDNA using the RevertAid First Strand cDNA Synthesis Kit according to the manufacturer’s instructions. The expression of mRNA levels was measured using a SYBR Mastermix (PowerUp SYBR Green Master Mix (Thermo Fisher Scientific) in a StepOnePlus Real-Time PCR machine (Applied Biosystems). The primers used for amplifying G6PD, Bax, and Bcl-2 cDNA were: G6PD forward 5′-GTCAAGGTGTTGAAATGCATC-3′ and reverse 5’-CATCCCACCTCTCATTCTCC-3’, Bax forward 5’-AACATGGAGCTGCAGA GGAT-3’ and reverse 5’-CAGCCCATGATGGTTCTGAT-3’, Bcl2 forward 5’-GGTGGGGTCATGTGTGTG-3’ and reverse 5’- CGGTTCAGGTACTCAGTCATC-3’, with β-actin as a reference gene, forward 5’-ACTCTTCCAGCCTTCCTTC-3’ and reverse 5’-ATCTCCTTCTGCATCCTGTC-3’. For qRT-PCR, fold changes in mRNA expression were calculated using the 2-ΔΔCt method with β-actin as the reference gene. Statistical analysis was performed as described in the Statistical analysis section.

### ROS measurement by CM-H2DCFDA assay

Cells were seeded in black 96-well plates at 5 × 10^3^ cells per well and allowed to attach overnight. Following attachment, cells were treated with various concentrations of 1,2-NQ derivatives for 48 h. After treatment, the medium was replaced with fresh medium containing 10 µM chloromethyl derivative of 2′,7′-dichloro-dihydro-fluorescein diacetate (CM-H2DCFDA, Thermo Fisher Scientific catalog No. C6827) and incubated for 30–60 min at 37 °C in the dark. Intracellular ROS were detected by the oxidation of CM-H2DCFDA to a fluorescent adduct, which was measured at 520 nm after excitation at 488 nm using a Synergy HT microplate reading fluorometer (BioTek Instruments).

### Cell-cycle analysis

Cells were seeded in 25 cm^2^ flasks at a density of 8.75 × 10^4^ cells and incubated overnight. The following day, cells were treated with various concentrations of 1,2-NQ derivatives for 48 h. Cell-cycle analysis was performed using a Beckman Coulter DxFlex flow cytometer following the manufacturer’s protocol. After treatment, cell pellets were fixed in 70% eagent-grade ethanol for 30 min at 4 °C. The cells were then stained with 50 µg/ml propidium iodide (ImmunoTools) and 100 µg/ml RNase (ThermoFisher Scientific) for 20 min at 4°C in the dark. Data analysis was conducted using CytExpert software (Version 2.0.2.18), provided by Beckman Coulter.

### Annexin-V and propidium iodide (annexin-V/PI) assay

Cell apoptosis was assessed using the FITC Annexin V Apoptosis Detection Kit (BD Pharmingen, Cat. No. 556547) following treatments with SJ006, DHEA, and 6-AN. Briefly, A549 and H292 cells were treated with 20 µM of each compound for 48 h. After treatment, the cells were harvested and resuspended in 500 µl of staining buffer, supplemented with propidium iodide (PI) and Annexin-V-FITC, according to the manufacturer’s instructions. The proportion of apoptotic cells was quantified using a Beckman Coulter DxFlex flow cytometer, and data were analyzed using CytExpert software (Version 2.0.2.18), provided by Beckman Coulter.

### Statistical analysis

All statistical analyses were performed using IBM SPSS Statistics for Windows (version 29). Nonlinear regression analysis was conducted to determine effective concentration (CC_50_) using the CC_50_ calculator program and Prism 10 (GraphPad Software). One-way ANOVA was used to compare cell viability, mRNA and protein expression, oxidative stress, and cell cycle arrest between the control group (no compound) and each treatment group (individual compounds) under the same experimental conditions. Where significant differences were detected, LSD post‑hoc tests were performed to identify pairwise differences. For single-treatment comparisons (e.g., DHEA or 6‑AN) versus control, one‑sample Student’s t‑tests were applied. Data are presented as mean ± SEM from three independent experiments (biological replicates), each with three technical replicates. Error bars represent SEM, and statistical significance was set at *p* < 0.05.

## Supplementary Information

Below is the link to the electronic supplementary material.

Supplementary Material 1

## Data Availability

No datasets were generated or analysed during the current study.
