# Supplementary material for: Methylated 1,2-naphthoquinone derivative SJ006 as an inhibitor of human glucose 6-phosphate dehydrogenase in non-small cell lung cancer cell lines
Source: Sci Rep. 2025 Oct 21;15:36746. doi: 10.1038/s41598-025-20702-6 (PMC12540661; doi:10.1038/s41598-025-20702-6)
Supplement: Supplementary file 1 — Supplementary Material 1 [file 41598_2025_20702_MOESM1_ESM.pdf]

Methylated 1,2-naphthoquinone derivative SJ006 as an inhibitor of human glucose 6-phosphate dehydrogenase in non-small cell lung cancer cell lines

Makamas Chanda, Warinthorn Chavasiri, Panupong Mahalapbutr, Thanyada Rungrotmongkol, Poonlarp Cheepsunthorn, Chalisa Louicharoen Cheepsunthorn

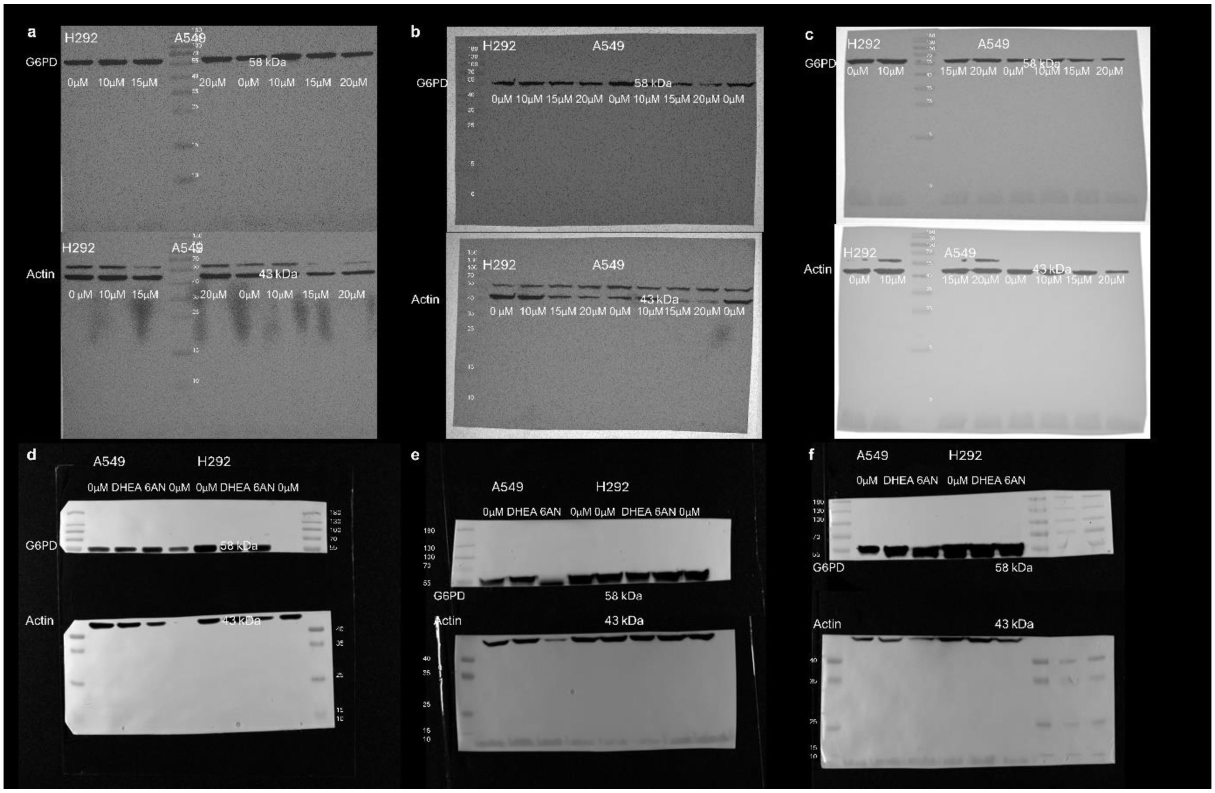

Supplementary Figure S1 Effects of SJ006 on G6PD protein expression in A549 and H292 cells. Western blot (WB) analysis of G6PD protein levels following treatment with SJ006 (3 independent replicates: a, b, c), DHEA, and 6-AN (3 independent replicates: d, e, f). Data are representative of three independent experiments.

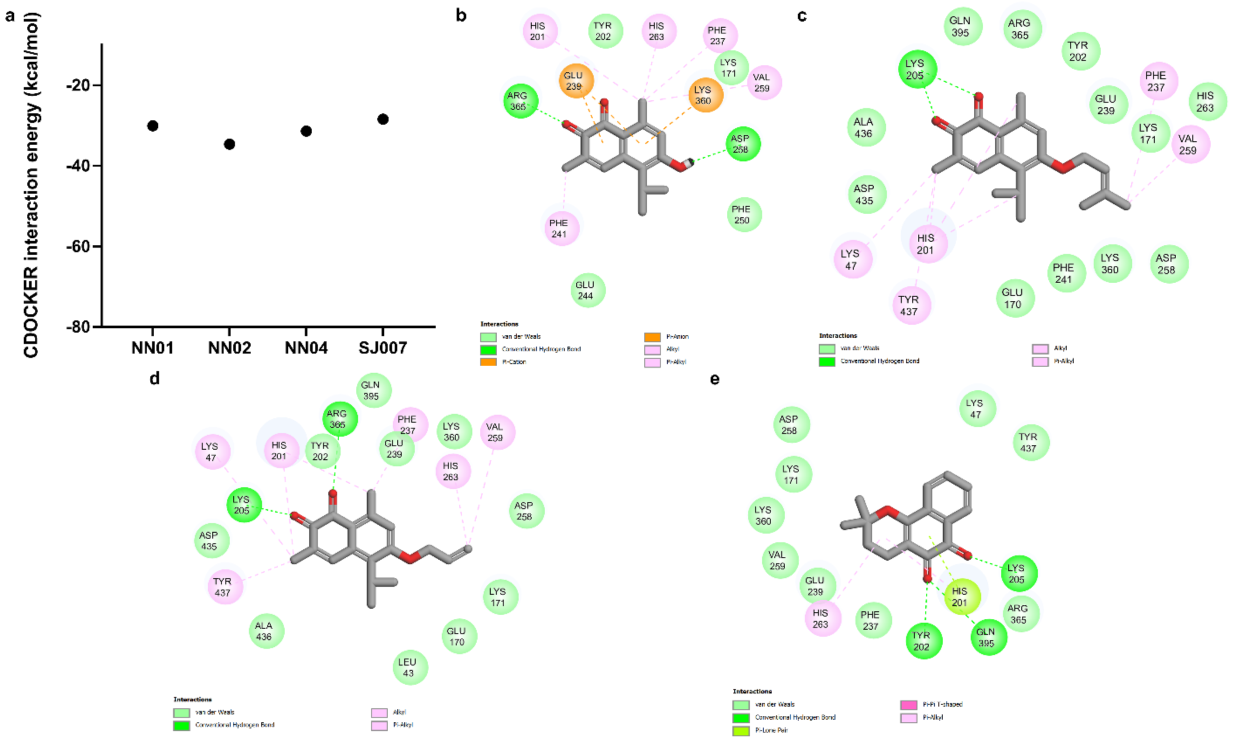

Supplementary Figure S2 Molecular docking energies and 2D interaction profiles of G6PD with NN01, NN02, NN04, and SJ007. (**a**) Comparison of molecular docking energies of NN01, NN02, NN04, and SJ007. 2D interaction profiles illustrating binding interactions of G6PD with (**b**) NN01, (**c**) NN02, (**d**) NN04, and (**e**) SJ007, highlighting key residues involved in hydrogen bonding, van der Waals forces, alkyl interactions, and other binding interactions.

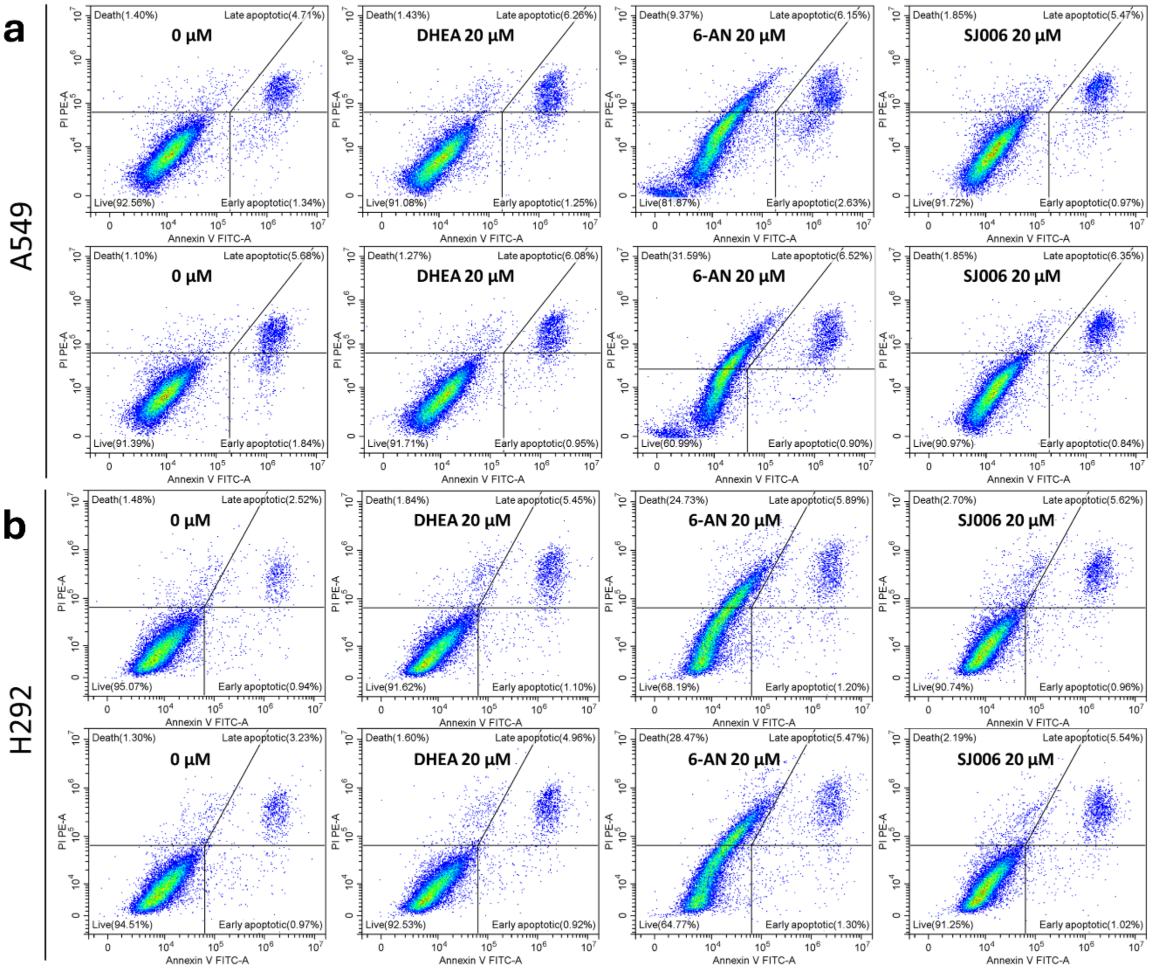

Supplementary Figure S3 Effects of SJ006 on apoptosis via G6PD inhibition in A549 and H292 cells assessed by annexin V/PI staining. Viable (lower left quadrant), early apoptotic (lower right quadrant), necrotic (upper left quadrant), and late apoptotic (upper right quadrant) cell populations in control (0 µM), DHEA, 6-AN, and SJ006 treatments in (**a**) A549 and (**b**) H292 cells. Data are presented as mean ± SEM.
